# Congenital factor V deficiency from compound heterozygous mutations with a novel variant c.2426del (p.Pro809Hisfs^∗^2) in the *F5* gene: A case report

**DOI:** 10.1097/MD.0000000000018947

**Published:** 2020-01-31

**Authors:** Chang-Hun Park, Min-Seung Park, Ki-O Lee, Sun-Hee Kim, Young Shil Park, Hee-Jin Kim

**Affiliations:** a Department of Laboratory Medicine and Genetics, Samsung Changwon Hospital, Sungkyunkwan University School of Medicine, Changwon; b Department of Laboratory Medicine and Genetics, Samsung Medical Center, Sungkyunkwan University School of Medicine; c Samsung Biomedical Research Institute, Samsung Medical Center; d Department of Pediatrics, Kyung Hee University Hospital at Gangdong, Seoul, Korea.

**Keywords:** case report, *F5*, factor V deficiency, mutation

## Abstract

**Introduction:**

Congenital factor V deficiency (FVD) is a rare bleeding disorder characterized by low or undetectable plasma factor V (FV) levels leading to mild to severe bleeding symptoms. Currently, more than 100 mutations have been reported in *F5*. We herein report a patient with FVD from mutations in the *F5* gene.

**Patient concerns:**

A 52-year-old man with prolonged prothrombin time and activated partial thromboplastin time corrected by mixing test on preoperative screening. His past medical or family history was not remarkable.

**Diagnosis:**

Factor assays revealed a markedly reduced FV activity at 7%. Other factors were not decreased. DNA sequencing analysis to detect *F5* gene mutations showed the patient was compound heterozygous for c.286G>C (p.Asp96His) and c.2426del (p.Pro809Hisfs∗2). Asp96His was previously described missense mutation and Pro809Hisfs∗2 was a novel deleterious mutation.

**Interventions:**

Fresh-frozen plasma was administered to supplement FV before surgery.

**Outcomes:**

Subsequent factor assays revealed temporarily increased FV activity at 33%.

**Conclusion:**

As was the case in our patient, genotype-phenotype correlations are poor in FVD, and molecular genetic test is necessary to confirm the diagnosis.

## Abstract 2

Supplemental Digital Content is available in the text

## 1 Introduction

Coagulation Factor V (FV) is a high-molecular weight glycoprotein (330 kDa) that plays a critical role in hemostasis by modulating the generation of thrombin. FV is composed of 6 domains, A1, A2, B, A3, C1, and C2.^[1–3]^ The gene encoding human FV is *F5*, which is located on the long arm of chromosome 1 (1q24.2) and consists of 25 exons spanning about 80 kb. Activation of FV is mediated by thrombin or the active form of factor X with cleavage at 3 arginine residues (Arg709, Arg1018, and Arg1545) and subsequent removal of the protruding B domain.^[4]^ FV is inactivated by cleavages at Arg306, Arg506, Arg679, and Lys994.^[4]^ Congenital factor V deficiency (FVD) is 1 of rare bleeding disorders (RBD) and is characterized by low or undetectable plasma FV levels. Clinically, bleeding symptoms in FVD is variable, from mild to severe.^[5,6]^ Bleeding symptoms include mucosal tract bleeding, epistaxis, oral cavity hemorrhages, menorrhagia, hemarthrosis, posttraumatic and postoperative bleeding. Life-threatening bleedings such as central nervous system hemorrhage are not common.^[6]^ Currently, more than 100 mutations have been reported in *F5* (Human Gene Mutation Database professional version, release 2018.3, last access January 16, 2019). The majority of *F5* mutations are missense mutations (61.5%), and small deletion and splicing mutations account for 18.5% and 11.9%, respectively.

In this report, we describe a patient with hereditary FVD from compound heterozygous mutations including a novel deleterious mutation.

## 2 Case report

### 2.1 Patient

A 52-year-old man was referred to the coagulation clinic of Samsung Medical Center because of prolonged prothrombin time (PT)/activated partial thromboplastin time (aPTT) on preoperative screening tests. He had avascular necrosis of both femoral heads that required surgical treatment. He had no history of bleeding symptoms. There was no familial history of bleeding. Coagulation tests demonstrated significantly prolonged PT (2.29 INR) and aPTT (78.3 second) (reference range, 29.1–41.9 second), both of which were corrected on mixing with normal plasma, indicating coagulation factor deficiency. Subsequent factor assays revealed a significantly decreased FV activity at 7% (reference range, 81%–160%). The results of other factor assays, II, VII, VIII, IX, X, XI, and XII, were not decreased.

### 2.2 Molecular genetic analysis

On suspicion of FVD, molecular genetic test was performed after obtaining written informed consent for publication of the case report and accompanying images. Genomic DNA was extracted from peripheral blood leukocytes, and direct sequencing analysis was performed to detect point mutations of *F5* covering all coding exons and flanking intronic regions by using primers developed by the authors (see Supplementary Table, which demonstrates primer sequences of *F5* gene) on ABI Prism 3130xl Genetic Analyzer (Applied Biosystems, Foster City, CA) with the BigDye Terminator Cycle Sequencing Ready Reaction kit (Applied Biosystems). The sequence chromatograms obtained were compared with the reference sequence of *F5* (NM_000130.4), and identified mutations were described following the recommendations by the Human Genome Variation Society (http://www.hgvs.org/mutnomen/) and classified according to the ACMG/AMP guidelines with reference to following public databases^[7]^: Human Gene Mutation Database (HGMD, http://www.hgmd.cf.ac.uk/ac/index.php), dbSNP (http://www.ncbi.nlm.nih.gov/projects/SNP/), 1000 Genomes (http://browser.1000genomes.org/), NHLBI Exome Sequencing Project (http://evs.gs.washington.edu/EVS/), and Exome Aggregation Consortium (http://exac.broadinstitute.org/). Genetic variants were assessed by pathogenicity prediction tools (Align GVGD, SIFT, PolyPhen2 and MutationTaster softwares). The websites were simultaneously consulted using Alamut Interactive Biosoftware.

**Figure 1:**
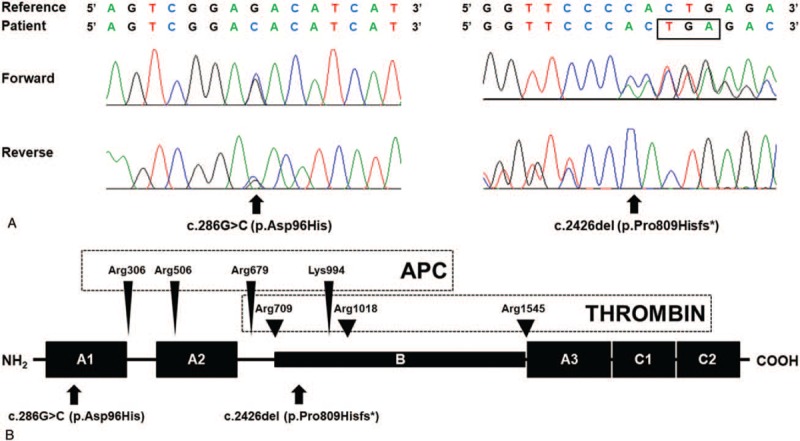
Molecular analyses of the *F5* gene in the patient with congenital factor V deficiency. (A) He was compound heterozygous for c.286G>C (p.Asp96His) (left panel) and c.2426del (p.Pro809Hisfs^∗^) (right panel; box indicates stop codon). (B) The location of the mutations in the factor V protein. The arrows indicate the mutations in the patient. The arrowheads indicate the cleavage sites of activated protein C (APC) and thrombin. APC = activated protein C.

As a result, 2 different heterozygous mutations in *F5* were detected: a missense variant (c.286G>C [p.Asp96His]) and a frameshift variant from small deletion (c.2426del [p.Pro809Hisfs∗2]) (Fig. 1A). Asp96His (Asp68His in the mature protein) was previously described in patients with FVD. Pro809Hisfs∗2 has not been listed in the public databases. According to the ACMG/AMP guidelines,^[7]^ Asp96His and Pro809Hisfs∗2 were a likely pathogenic variant and a pathogenic variant causing FVD, respectively.

Before surgical treatment for avascular necrosis of both femoral heads, fresh-frozen plasma (FFP) was administered to supplement FV. Subsequent factor assays revealed temporarily increased FV activity at 33%. FFP was transfused for 5 days after surgery, and peri- and postoperative course was uneventful.

## 3 Discussion

FVD from homozygous or compound heterozygous mutations usually have FV levels lower than 10%, whereas those from a single heterozygous mutations have FV levels typically around 50%.^[5]^ In our patient, the FV level was less than 10%, which suggested a homozygous or compound heterozygous mutation state. Genetic study of *F5* revealed compound heterozygosity of Asp96His, a known mutation, and Pro809Hisfs∗2, a novel deleterious mutation. Although we could not perform family study to determine the allelic status of 2 mutations, compound heterozygous status was inferred by the residual FV activity (7%). Asp96His and Pro809Hisfs∗2 are located in the A1 domain and the B domain, respectively (Fig. 1B). Pro809Hisfs∗2 from c.2426del is predicted to cause frameshift and thereby truncated protein. It is known that most missense mutations of *F5* cluster in A2 and C2 domains. According to HGMD, approximately 20% of *F5* mutations are located in the B domain, which is disposed during the enzymatic activation of FV. Asp96His was reported to cause disruption of salt bridge and subsequent interference in protein expression.^[8–10]^ Interestingly, all previously reported cases with FVD from Asp96His of *F5* were of Asian descent (1 Korean and 4 unrelated Chinese patients).^[8–10]^ According to the genome aggregation database (http://gnomad.broadinstitute.org/), the population frequency of Asp96His was 0.001105 in East Asians, while the variant is not observed in other populations. This suggests that Asp96His could be a founder mutation with shared genetic backgrounds among Asians.

The prevalence of FVD is 1:1000 and 1:1 000 000 in heterozygotes and homozygous forms, respectively, accounting for 8.3% of all RBDs.^[5,6]^ According to the 2017 Annual Report of Korea Hemophilia Foundation, RBDs represent ∼4% of bleeders in Korea, including 0.3% with FVD. Recently, the European Network of Rare Bleeding Disorders suggested categories of bleeding severity from asymptomatic to grade I/II/III bleeding.^[11]^ The association between the clinical severity and residual factor activities has been variably reported in RBDs, with a poor correlation in FVD.^[12]^ In our previous study, we even observed a notable intrafamilial variability of bleeding symptoms in patients and their families with the same genotype.^[10]^ In line with the previous reports, the patient described in this report had a very low FV activity of 7% and was compound heterozygous mutations, but had experienced no bleeding symptoms. Thus, given the variety of acquired conditions associated with decreased FV activities, mutation identification is necessary to confirm FVD irrespective of clinical symptoms.

Since FV-containing concentrates are not available and FV is not contained in cryoprecipitate or prothrombin complex concentrates, the clinical management of FVD is primarily based on replacement therapy with FFP.^[5]^ In our case, FFP was administered to supplement FV before joint surgery.

In summary, we herein report an asymptomatic patient with FVD from compound heterozygous mutations of *F5* including a novel frameshift mutation. As was the case in our patient, genotype-phenotype correlations are poor in FVD, and molecular genetic test is necessary to confirm the diagnosis.

## Author contributions

**Conceptualization:** Young Shil Park, Hee-Jin Kim.

**Data curation:** Chang-Hun Park.

**Formal analysis:** Min-Seung Park, Ki-O Lee.

**Investigation:** Chang-Hun Park, Min-Seung Park, Sun-Hee Kim.

**Methodology:** Min-Seung Park, Ki-O Lee.

**Project administration:** Hee-Jin Kim.

**Resources:** Chang-Hun Park, Sun-Hee Kim, Young Shil Park.

**Software:** Sun-Hee Kim.

**Supervision:** Young Shil Park, Hee-Jin Kim.

**Validation:** Chang-Hun Park, Min-Seung Park, Ki-O Lee, Young Shil Park, Hee-Jin Kim.

**Visualization:** Chang-Hun Park, Min-Seung Park.

**Writing – original draft:** Chang-Hun Park.

**Writing – review & editing:** Hee-Jin Kim.

Chang-Hun Park orcid: 0000-0002-0412-3732.

## Supplementary Material

Supplemental Digital Content
